# Glutathione is key to the synergistic enhancement of doxorubicin and etoposide by polyphenols in leukaemia cell lines

**DOI:** 10.1038/cddis.2015.379

**Published:** 2015-12-31

**Authors:** A A Mahbub, C L Le Maitre, S L Haywood-Small, N A Cross, N Jordan-Mahy

**Affiliations:** 1 Biomolecular Sciences Research Centre, The Department of Biosciences and Chemistry, Faculty of Health and Wellbeing, Sheffield Hallam University, Howard Street, Sheffield, South Yorkshire S1 1WB, UK

Recently published in *Nature: Cell Death and Discovery*, Mahbub *et al.*^1^ have demonstrated that polyphenols can synergistically enhance the action of the topoisomerase II inhibitors: doxorubicin and etoposide in leukaemia cells. A reduction of glutathione (GSH) was strongly associated with sensitising cells to the pro-apoptotic effects of polyphenols when used in combination with doxorubicin or etoposide. Importantly, when polyphenols and topoisomerase II inhibitors were combined, it was possible to induce a synergistic decrease in cell proliferation (measured as ATP levels), cell-cycle arrest and induction of apoptosis in leukaemia cell lines.^1^

Five polyphenols that had been previously shown to induce apoptosis in leukaemia cells (quercetin, apigenin, emodin, rhein and *cis-*stilbene)^2^ were combined with doxorubicin or etoposide in two lymphoid (CCRF-CEM and Jurkat) and two myeloid (THP-1 and KG1a) cell lines. These cell lines were selected as they had been identified as the most sensitive and most resistant to polyphenol-induced apoptosis;^2^ in addition, two non-tumour control haemopoietic stem cells (HSCs) (CD133+ and CD34+) were investigated.

In the two lymphoid cell lines, it was shown that all studied polyphenols when used in combination with each topoisomerase II inhibitor caused a synergistic or additive decrease in cell proliferation, G_2_M or S phase cell-cycle arrest and apoptosis. This was associated with a synergistic/additive reduction of GSH levels, increased caspase 3, 8 and 9 activity, and DNA damage (Figures 1a and b). In the non-tumour control HCS cells the polyphenols had a protective effect; following combination treatment with the topoisomerase II inhibitors there was an increase in cell proliferation and a decrease in apoptosis.

In myeloid cell lines there was a more differential effect: when quercetin and apigenin were used in combination with each topoisomerase II inhibitor, there was a synergistic/additive decrease in cell proliferation, cell accumulation in G_2_M and S phase of the cell cycle and an increase in apoptosis. This was associated with decreased GSH levels, increased caspase 3, 8 and 9 activity, and DNA damage (Figure 1c). However, when emodin, rhein and to a lesser extent *cis*-stilbene, were used in combination with each topoisomerase II inhibitor, there was an antagonistic increase in ATP, an inhibition of apoptosis and no cell-cycle arrest. This was associated with an elevation of GSH levels and reduction of caspase 3, 8 and 9 activity, and little or no DNA damage (Figure 1d).

Examination of basal GSH levels showed that the levels in the lymphoid leukaemia cell lines were significantly lower than those of the non-tumour control HSCs and myeloid cell lines. This could explain why the lymphoid cell lines are more susceptible to polyphenol/topoisomerase II inhibitor treatment compared with the myeloid cell lines. The identification of GSH as a key player in the induction of apoptosis was first highlighted by Franco and Cidlowski in 2009.^3^ Initially, GSH depletion was considered as a by-product of ROS production during mitochondrial permeability during apoptosis via the intrinsic route;^3^ however, it is now clear that although mitochondrial ROS formation is crucial for apoptosome formation;^4^ reduced GSH is necessary for normal cells to undergo apoptosis, independently of ROS.^5^ This led to the suggestion that polyphenol-mediated decrease in intrinsic GSH or efflux can sensitise lymphoid cancer cell lines to topoisomerase II inhibitors, which results in the synergistic and additive induction of apoptosis.

Although our *in vitro* studies in lymphoid leukaemia cell lines suggest that the combination of polyphenols and topoisomerase II inhibitors may selectively enhance antitumour effects in tumour *versus* normal HSCs, future *in vivo* studies are needed to confirm that these effects are maintained *in vivo*. Where enhanced activity is observed, translational studies need to establish that enhanced activity is not counterproductive in terms of just increasing drug potency and its associated side effects, and hence no antitumour benefit.

In contrast, the differential effects seen in the myeloid cell lines to polyphenol/topoisomerase II inhibitor combination treatments can be attributed to changes in GSH levels. Where myeloid cells were treated with quercetin and apigenin in combination with topoisomerase II inhibitors, this caused a depletion of GSH levels and the induction of apoptosis; although antagonism of doxorubicin and/or etoposide was observed with emodin, rhein and *cis*-stilbene, this occurred in the presence of unchanged or elevation of GSH levels. This supports the idea that the elevation of GSH is linked to chemotherapy resistance. Indeed high GSH levels are commonplace in many cancer cells,^6, 7^ and efflux of GSH is considered to be key in the development of multi-drug resistance.^8^ Furthermore, in myeloid leukaemia patients, GSH levels and its associated enzyme GSH transferase are commonly high, and are associated with increased risk of disease relapse and resistance to chemotherapy treatments.^9^

The observed polyphenol-mediated antagonism of topoisomerase II inhibitors in myeloid leukaemia cells is especially important as polyphenols are abundant in a balanced diet. This antagonistic effect of polyphenols has been previously observed by Lui *et al.*,^10^ who showed that quercetin antagonised the proteasome inhibitor bortezomib (Velcade) in breast cancer cell lines, and raised the potential negative effect of a polyphenol-rich diet with certain antitumour regimes. The results of Mahbub *et al.*^1^ raise the possibility of a similar effect in myeloid malignancies treated with topoisomerase II inhibitors, in that dietary polyphenols may prevent etoposide/doxorubicin-induced antitumour activity. The mechanism of action of polyphenol-mediated antagonism of topoisomerase II inhibitors is unclear. However, it is known that GSH is contra-indicated for other chemotherapy agents, such as for cisplatin, where GSH supplementation inhibits the action of cisplatin. However, GSH-mediated depletion appears unrelated to cisplatin insensitivity in myeloid leukaemia cell lines.^11^ This is, however, in contrast to most other tumour models, suggesting that alternate multi-drug resistance mechanisms may be a feature of myeloid leukaemia cell lines. Similarly, recent work has shown that antioxidants can increase the metastasis of melanoma in mice,^12^ which raises the possibility that in some cancer types, polyphenols and other antioxidants could be detrimental. Thus, it is fundamental to tailor any treatment, be it with novel antitumour agents such as polyphenols or standard chemotherapy, to the specific cancer types and investigate any possible treatment interactions.

## Figures and Tables

**Figure 1:**
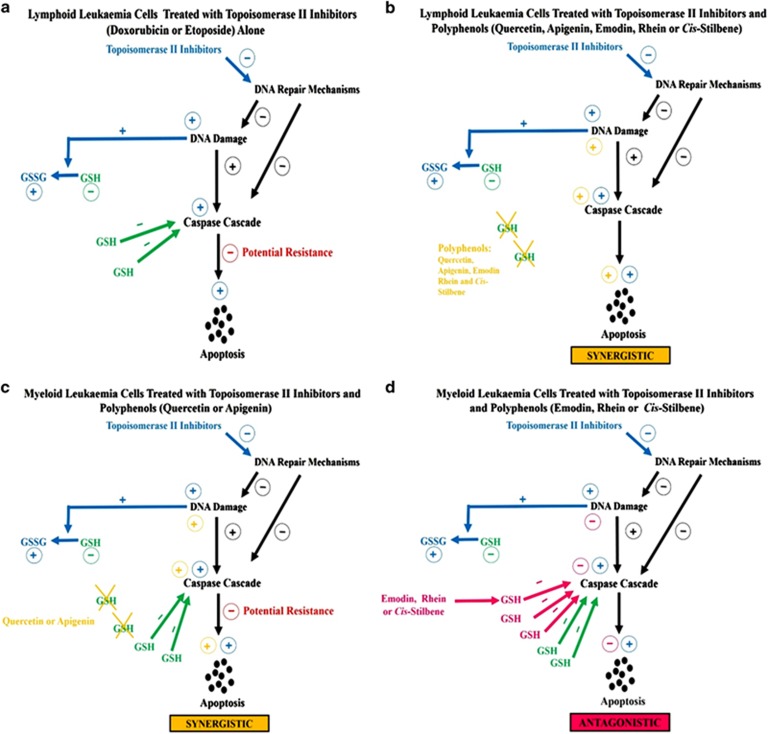
A schematic showing the effects of topoisomerase II Inhibitors (doxorubicin or etoposide) alone and in combination with polyphenols (quercetin, apigenin, emodin, rhein or *cis*-Stilbene) in lymphoid and myeloid leukaemia cells
